# The Causal Cascade to Multiple Sclerosis: A Model for MS Pathogenesis

**DOI:** 10.1371/journal.pone.0004565

**Published:** 2009-02-26

**Authors:** Douglas S. Goodin

**Affiliations:** Department of Neurology, University of California San Francisco, San Francisco, California, United States of America; University of East Piedmont, Italy

## Abstract

**Background:**

MS pathogenesis seems to involve both genetic susceptibility and environmental risk factors. Three sequential factors are implicated in the environmental risk. The first acts near birth, the second acts during childhood, and the third acts long thereafter. Two candidate factors (vitamin D deficiency and Epstein-Barr viral infection) seem well suited to the first two environmental events.

**Methodology/Principal Findings:**

A mathematical Model for MS pathogenesis is developed, incorporating these environmental and genetic factors into a causal scheme that can explain some of the recent changes in MS-epidemiology (e.g., increasing disease prevalence, a changing sex-ratio, and regional variations in monozygotic twin concordance rates).

**Conclusions/Significance:**

This Model suggests that genetic susceptibility is overwhelmingly the most important determinant of MS pathogenesis. Indeed, over 99% of individuals seem genetically incapable of developing MS, regardless of what environmental exposures they experience. Nevertheless, the contribution of specific genes to MS-susceptibility seems only modest. Thus, despite HLA DRB1*1501 being the most consistently identified genetic marker of MS-susceptibility (being present in over 50% of northern MS patient populations), only about 1% of individuals with this allele are even genetically susceptible to getting MS. Moreover, because genetic susceptibility seems so similar throughout North America and Europe, environmental differences principally determine the regional variations in disease characteristics. Additionally, despite 75% of MS-patients being women, men are 60% more likely to be genetically-susceptible than women. Also, men develop MS at lower levels of environmental exposure than women. Nevertheless, women are more responsive to the recent changes in environmental-exposure (whatever these have been). This explains both the changing sex-ratio and the increasing disease prevalence (which has increased by a minimum of 32% in Canada over the past 35 years). As noted, environmental risk seems to result from three sequential components of environmental exposure. The potential importance of this Model for MS pathogenesis is that, if correct, a therapeutic strategy, designed to interrupt one or more of these sequential factors, has the potential to markedly reduce or eliminate disease prevalence in the future.

## Introduction

Human diseases such as multiple sclerosis (MS) are often chronic and have complex etiological bases [1]. Both the genetic background and environmental events are critical. For example, an individual from northern Europe or northern North America has a life-time MS risk of about 1–2 per 1,000 population [2]. Risk for individuals with an affected family member increases in proportion to the shared genetic information between themselves and the proband [2]–[8]. Monozygotic-twins of an MS proband have approximately 200 times the risk in the general population [2]–[8]. Nevertheless, it is clear that genes are not the only disease determinants. Otherwise, the proband-wise concordance-rate for monozygotic-twins would be closer to 100% than the reported 20–30% in these northern populations [3], [9]–[11]. This conclusion is even more apparent in southern populations where the concordance-rate for monozygotic-twins is approximately half that in the north [11]–[13]. Consequently, that there must be important environmental or epigenetic factors involved in MS pathogenesis. Although the present paper explores, through a mathematical Model, the relationship of genetic predisposition and environmental exposure to recent changes in MS epidemiology, in many respects, the Model is also applicable to other complex human diseases.

### The Nature of the Environmental Exposure

Important observations regarding MS pathogenesis relate to the absence of micro-environmental contributions to MS risk. Thus, studies in adopted individuals, conjugal couples, brothers and sisters of different birth order, and in siblings and half-siblings raised together or apart, have demonstrated that micro-environmental influences don't contribute measurably to MS risk [4], [7], [14]–[17]. Consequently, the relevant environmental events must act at a population level. Moreover, if, in addition to a genetic predisposition, one or more population level environmental events need to occur in order for MS to develop, then it is only natural to enquire as to how many such events are necessary and whether it is important for these events to occur at any particular time or in any particular order. Three sets of epidemiological observations bear on these issues.

#### A Very Early Environmental Influence

The first set relates to a maternal effect in MS [7]. This maternal effect is supported by three epidemiological observations. The first is that half-siblings, who are concordant for MS, are twice as likely to share the mother compared to the father [4], [7], indicating that MS susceptibility is being passed from mother to child through something other than nuclear genes. An environmental exposure, occurring either in the intrauterine or in the early post-natal period, is one possibility. After the child becomes independent of their mother, however, such a maternal effect would be unexpected from an environmental factor.

This maternal effect, however, need not be environmental. It could also be due to mitochondrial genes, from genetic-imprinting favoring expression of certain maternal genes, or from other epigenetic factors [18]. With respect to these other possibilities, there has been an interesting debate about the existence of a so-called “Carter effect” in MS, whereby paternal transmission would be favored for certain genetic traits [19], [20]. One report found weak evidence (p = 0.032) for preferential paternal transmission of MS risk [19] whereas a larger Canadian study found no such effect [20]. Importantly, however, neither study showed the preferential maternal transmission expected if mitochondrial genes, genetic imprinting, or epigenetic factors explained the maternal effect in MS [7]. An environmental cause for the maternal effect, by contrast, would not be observed in these studies because the maternal intra-uterine and post-natal environments are the same regardless of which parent transmits MS risk.

The second observation is that the MS concordance-rate for fraternal-twins seems to be greater than that for full-siblings [6], [10]. Thus, in the large Canadian cohort [10], the MS concordance-rate in full-siblings was reported to be 2.9% with a standard-error (SE) of 0.6%, compared to the concordance-rate in dizygotic-twins, which was 5.4% (no statistical comparison provided). A recent review [6] reached the same conclusion. Such a disparity cannot be explained by mitochondrial inheritance, genetic imprinting, or epigenetic factors because, on average, these should be similar for siblings and fraternal twins sharing the same biological parents. Rather, this discrepancy must be due to environmental factors acting during the shared intra-uterine or early post-natal period.

The third observation relates to the month-of-birth effect in MS reported in Canada and northern Europe [21]–[23]. In a study combining patients from Canada, Denmark, and Sweden, significantly more MS patients were born in May and fewer in November, compared to background rates during the rest of the year [21]. Another study found more RRMS patients born in May than November [23]. Finally, in 67 Canadian patients, born in the southern hemisphere, this month-of-birth effect seemed reversed [21]. Whether such a reversal is generally found in the southern hemisphere must await further study.

These observed month-of-birth effects provide clear evidence for an early environmental influence, involved in MS pathogenesis, time-locked to birth. As the time interval between the birth and any environmental event increases, the coupling between birth and the event should become increasingly less precise and, thus, the birth-signal should become increasingly less distinct. The fact that this signal is so clear [21], suggests that the relevant environmental event occurs very near to the birth itself. Moreover, this environmental event is periodic and appears coupled to the solar cycle [21]. Of possible relevance to this circa annum periodicity for MS susceptibility, is the fact that mothers of May babies (who spend much of their intrauterine life during the winter months) will experience less sun exposure during their pregnancies compared to mothers of November babies. It is tempting to speculate (and has been speculated), therefore, that this maternal environmental event is related to a low maternal sun-exposure (perhaps resulting in low levels of vitamin D) while the child is *in utero* [21], [24].

#### Childhood/Adolescent Environmental Influences

The second set of observations relates to an environmental influence in persons who migrate from one region to another [2], [25]–[29]. When individuals move (prior to approximately age 15 years) from a high MS prevalence area to a low prevalence region (or vice versa), their MS risk is similar to that of the region to which they moved. By contrast, when they make the same move after age 15, their MS risk is similar to that of the region from which they moved. Similarly, children of immigrants from low prevalence regions (born in high-prevalence areas) have an MS risk similar to their birth country rather than their country of ethnic origin [25]. These observations implicate an environmental event, involved in MS pathogenesis, which acts sometime between birth and young adulthood (∼15 years) but does not act thereafter.

#### Adult Environmental Influences

The third set of observations relate to the fact that the initial clinical symptoms in MS are typically delayed by many years (often decades) after this critical early-period when the maternal factor and the migratory factor operate [2], [25]–[29]. Perhaps, only these critical early events are necessary although the apparently low MS penetrance is difficult to explain, at least with respect to some candidate environmental factors. Thus, despite the fact that Epstein-Barr viral (EBV) infection, especially when it causes symptomatic mononucleosis, has been consistently linked to MS [30]–[38], fewer than one in 900 individuals with an EBV infection and only a small fraction of patients with mononucleosis will ever develop MS (Table 1). Consequently, it seems most likely that subsequent environmental events dictate the timing of symptom onset.

**Table 1:** Prevalence of antibodies to EBV in the sera of patients and controls.

Study	EBV+ MS Cases (%)	EBV+ Controls (%)	p value
Sumaya, 1980 [73] ‡	155/157 (98.7%)	76/81 (93.8%)	0.05
Bray, 1983 [74] ‡	309/313 (98.7%)	363/406 (89.4%)	0.0001
Larson, 1984 [75] ‡	93/93 (100%)	78/93 (83.9%)	0.0001
Sumaya, 1985 [76] *	104/104 (100%)	23/26 (88.5%)	0.007
Shirodaria, 1987 [77] ‡‡	26/26 (100%)	24/26 (92.3%)	-
Munch, 1998 [78] †	137/138 (99.3%)	124/138 (89.9%)	0.0004
Myhr, 1998 [79] *	144/144 (100%)	162/170 (95.3%)	0.008
Wagner, 2000 [80] †	107/107 (100%)	153/163 (93.9%)	0.01
Ascherio, 2001 [30] ††	143/144 (99.3%)	269/287 (93.7%)	0.008
Sundström (2004) [32]	234/234 (100%)	693/702 (98.7%)	ns
Haahr, 2004 [81] †	153/153 (100%)	50/53 (94.3%)	0.05
Ponsonby, 2005 [31] ‡‡	136/136 (100%)	252/261 (96.6%)	0.05
**Total**	**1741/1749 (99.5%)**	**2267/2406 (94.2%)**	**p<10^−23^**

* Study measured antibodies against the Epstein-Barr nuclear antigens (EBNA), the viral capsid antigen (VCA), and the early antigens (EA).

‡ Study measured antibodies only against VCA.

† Study measured antibodies only against EBNA and EA.

†† Study measured antibodies only against EBNA and VCA. One person was antibody negative to each antigen but it is unclear from the text whether they were the same person. The review by Haahr in 2006 [81] suggests they were not.

‡‡ Study measured antibodies only against EBNA and VCA.

#### Causal Factors in MS Pathogenesis

Many factors, including small pox, typhoid, EBV, other infections, vitamin deficiencies, low-sunlight, cosmic-rays, occupational hazards, living with domesticated animals, dietary habits, trauma, stress, and toxic exposures have all been postulated to be linked to MS pathogenesis [2]. Of these possibilities, however, vitamin D deficiency and EBV infection have been gaining the greatest support for their potential role in MS pathogenesis. Moreover, each of these factors seem particularly well suited to the epidemiological scheme outlined above.

#### Vitamin D

The *in vivo* production of active vitamin D in humans requires the conversion of 7-dehydro-cholesterol into vitamin D_3_ [39]–[42]. The first of this two-step transformation (to pre-vitamin D_3_) is catalyzed by the exposure of 7-dehydro-cholesterol in the skin to ultraviolet B (UVB) radiation (i.e., electromagnetic radiation having a wavelength of 280–320 nm). Subsequently, stable Vitamin D_3_ is formed by an internal rearrangement of the double bond structure of the pre-vitamin D_3_ molecule. Vitamin D_3_ is then hydroxylated, firstly to 25(OH) D_3_ by 25-hydroxilase (mostly in the liver) and secondly by 1-α hydroxilase in the tissues (including the kidney) to form active vitamin D (1,25(OH)_2_D_3_). Dietary intake of vitamin D_3_ can bypass the UVB-dependent part of this pathway and permits maintenance of normal vitamin D serum levels, even in the absence of adequate UVB radiation. Unfortunately, however, there are few natural dietary sources of vitamin D_3_. It is only found in certain animals [43] such as oily fishes and reindeer, which derive it or its precursors from their diet (from phytoplanctonic algae in the case of fish and from lichen in the case of reindeer). Interestingly, two human populations with a notably low risk for MS [43]–[46] are the Inuit or Eskimos (who consume oily fish on a daily basis) and the Sami or Lapps (for whom reindeer meat is a staple). In both of these populations, their main source of active vitamin D comes from their diet [43]. For many other human populations, by contrast, exposure of the skin to the UVB radiation is of paramount importance for maintaining adequate active vitamin D levels throughout the year.

In this context, it is of considerable interest that, as latitude increases (both north and south of the equator), the amount of effective UVB radiation decreases due to an increase in the angle of the sunlight reaching the planet surface (i.e., traveling a longer distance through the Earth's atmosphere). In central and north-east Africa (where *Homo sapiens* evolved) plenty of UVB radiation is available for vitamin D synthesis throughout the year [47]–[49]. However, when humans migrated out of Africa approximately 50,000 years ago and began to inhabit the temperate regions of the globe, adequate UVB exposure throughout the year was no longer guaranteed. In fact, it has been estimated that the levels of UVB radiation at the latitude of the US-Canadian border are insufficient to produce an adequate amount of active vitamin D during most months of the year, especially in winter [47]–[49]. Moreover, as has been noted by many authors and consistent with a role for vitamin D deficiency in MS pathogenesis, world-wide distribution maps of reduced UVB availability are strikingly similar to world-wide distribution maps of MS prevalence [2], [47], [48], [50], [51].

Vitamin D acts in the body through binding to its receptor (VDR). Once such binding takes place, this ligand-receptor complex is internalized, forms a heterodimer with the retinoid X receptor (the nuclear receptor for 9-cis retinoic acid), and this heterodimer is translocated to the nucleus where it acts as a transcription factor, binding, often together with co-factors, to vitamin D response elements (VDREs) in the promoter region of several nuclear genes [41]. Interestingly, and perhaps very informatively, VDRE has recently been identified in the promoter region adjacent to the HLA DRB1*1501 allele, an allele and a portion of the genome that has been consistently linked to MS pathogenesis in Caucasian populations [2], [52]. The binding of vitamin D to the VDRE regulates the expression of multiple genes, acting either positively or negatively depending upon the specific gene involved.

The roles of vitamin D in calcium homeostasis and the maintenance of bone health are widely recognized [39], [42]. Less well known is its role in other processes, including anti-neoplastic actions and a variety of immune functions such as cell proliferation, differentiation, and immunomodulation [40], [41], [53]–[55]. Nevertheless, VDR is widely expressed throughout the body, including on activated T and B lymphocytes, and on macrophages [41], [53], [54] and vitamin D has been implicated in the maturation of dendritic cells and in the modulation of antigen-specific immune responses *in vivo* [53]–[55]. During gestation, human decidual cells synthesize active vitamin D, particularly in early pregnancy, and this has led to the suggestion that vitamin D may help to regulate both acquired and innate immune responses at the fetal-maternal interface [56]. Finally, vitamin D deficiency has been implicated in the likelihood of developing a variety of autoimmune diseases including insulin dependent diabetes mellitus, rheumatoid arthritis, experimental autoimmune encephalomyelitis (EAE), and inflammatory bowel disease [53], [54], [57]. Because these autoimmune diseases are more prevalent in females, this raises the possibility that there might be differences in the physiological responses to vitamin D between males and females and, in fact, such gender-specific differences in vitamin D metabolism have been reported [58], [59]. Interestingly, in one of these studies [59] vitamin D supplementation was found to confer protection against EAE only in intact female mice but not in males or in ovariectomized females.

As a result of these considerations, vitamin D deficiency seems to be a good candidate for the early (maternal) event noted above. Not only is this maternal factor (like vitamin D) coupled to the solar cycle in temperate regions [21], vitamin D is known to be involved in immune development and maturation [40], [41], [53]–[55], its deficiency is associated with autoimmunity [53], [54], [57], the world-wide distribution of reduced UVB radiation mirrors that of MS prevalence [2], [47]–[49], extreme northern populations with high dietary intake of vitamin D such as the Inuit and Sami people have a very low MS prevalence [43]–[46], and there are interactions between the physiological effects of vitamin D and gender in some mammals [58], [59]. This last aspect of vitamin D physiology might provide insight to why MS prevalence is gender-specific and, perhaps, also, to why MS incidence is increasing, especially among women [60]–[65].

#### Epstein-Barr Virus

EBV is a member of the herpes family of double-stranded linear DNA viruses, which is also referred to as the human herpes virus 4 (or HHV-4). It is one of the most common viral infections of humans, with over 90% of individuals becoming infected at some time during their life [36]. As maternal antibody protection disappears after birth, infants become susceptible to infection by EBV. In many parts of the world the initial EBV infection typically occurs during early childhood and is either asymptomatic or causes only mild symptoms indistinguishable from numerous other childhood infections. In developed regions such as the North America and Europe, however, the initial infection is often delayed until adolescence or young adulthood (possibly related to better hygiene), in which case infectious mononucleosis (glandular fever) develops in 35 to 50% of instances. The principal targets of the initial viral infection are the epithelial cells of the oropharynx and the B-lymphocytes.

Once a cell is infected, the viral genome becomes circularized and persists within the cell as an episome, executing distinct genetic programs, which result in either a lytic or a latent infection. Lytic infections produce a large number of free viral particles, which then can infect other B lymphocytes. Latent infection ultimately predominates (probably due to the host immune response) and the latent phase genetic programs cause the infected B-lymphocytes to proliferate and to be directed to the sites (e.g., the bone marrow) where the virus persists indefinitely. Such persistence is made possible by the virus turning off most of its genes, only occasionally to be reactivated, to produce fresh virions, and to cause further cell lysis.

With an acute infection, antibodies to antigens associated with viral replication, the viral capsid antigen (VCA) and the diffuse and restricted early antigens (EA), appear during either the late incubation period or the acute illness [66]. Antibodies to the VCA are initially of the IgM class. However, this response lasts only 1–2 months, after which the anti-VCA antibody response shifts to the IgG class. These antibodies persist for the lifetime of the individual. Antibodies to EA are of the IgG class and often drop to undetectable levels after 3–6 months. Nevertheless, although EA antibodies are a sign of active infection, approximately 20–30% of individuals will have persistent titers for years and these antibodies are also found in patients with chronic active infections or with secondary complications such as Burkitt's lymphoma or nasopharyngeal carcinoma. Antibodies to one or more of the EBV nuclear antigens (EBNA 1 to 5) that are expressed in latently infected B lymphocytes, appear 3–6 weeks after the initial infection. These antibodies persist for the lifetime of the individual. Other than MS, EBV infection has also been implicated as playing some role in the pathogenesis of certain malignancies (e.g., nasopharyngeal carcinoma, EBV-positive Hodgkin lymphoma, and Burkitt's lymphoma) as well as in several autoimmune disorders such as Sjogren's syndrome, rheumatoid arthritis, and systemic lupus erythematosus [67]. In these conditions, persistent titers to EBV are also found, suggesting that chronic immune activation against EBV may actually be participating in the pathophysiological process [67].

In contrast to vitamin D, however, EBV cannot be the maternal factor because infection does not typically occur either *in utero* or during the early post-partum period. Rather, EBV seems a much better candidate for the second environmental event. Nevertheless, vitamin D deficiency could also act during childhood and, indeed, some of the direct data supporting a role of vitamin D in MS pathogenesis actually suggests an effect in childhood or early adulthood [68]–[71]. Despite this, however, the evidence for EBV involvement in MS pathogenesis is compelling [30], [31], [34]–[38], [72]. Thus, even though EBV infection occurs in over 90% of populations matched for age and sex with MS patients [30]–[32], [73]–[81], the evidence for prior EBV infection in adult onset MS is essentially 100% and significantly more likely than controls (Table 1). Even in those rare MS patients who test negatively for prior EBV exposure, this result could easily be a false negative finding because, in every such case, the antibody response hasn't been measured to the entire set of EBV antigens (Table 1). Also, the prior nature of the EBV infection seems clear both by the presence of IgG (not IgM) antibodies to EBV antigens and by the unequivocal evidence (when it has been possible to measure) of infection long before the advent of clinical MS symptoms [10]–[32], [36], [37], [72]–[81]. Such a strong association is very hard to ignore.

Although this apparent high prevalence of prior infection in MS could conceivably be the consequence of either false negative tests within the general population or false positive tests in MS patients, both possibilities seem unlikely. Thus, the near 100% prevalence cannot be due to a general hyper-immune state in MS patients because their antibody responses to other common pathogens (e.g., cytomegalovirus, measles, mumps, chicken pox, herpes simplex, etc.) are not similarly increased [30]–[32], [74], [81]. Moreover, a recent pathological study found evidence of EBV infection in a substantial proportion of those B lymphocytes infiltrating the central nervous system in 21 of 22 MS cases examined at postmortem [82]. In addition, evidence of viral reactivation seemed to be restricted to ectopic B-cell follicles in the meninges and in acute lesions [82]. Finally, the increased risk of MS, either with delayed exposure to EBV [30], [31], [35], [81] or following symptomatic mononucleosis [32], [34], [35], [38], strongly suggests that the association between MS and this particular pathogen is genuine. Taken at face value, this near 100% association indicates that EBV is a necessary (but not a sufficient) condition for adult MS to develop and, therefore, that EBV must be a part of the causal pathway leading to MS. If EBV infection is permissive in this way then, like the second environmental factor in MS pathogenesis, it probably acts during childhood or adolescence (when both late infection and mononucleosis occur). There are no leading candidates for the third (or other) environmental factor or factors.

#### Changing Environmental Influences

It is also of note that MS epidemiology has changed in important ways over the past several decades. Thus, the incidence (prevalence) of MS is increasing [60]–[65], [83]–[86], especially in women [60]–[65]. As a consequence of this, the sex-ratio has been altered [65] and, recently, a switch in the latitude gradient for MS incidence has been reported [60]. Because MS genetics seems unlikely to have shifted in so short an interval, these observations presumably relate to a change in the environmental determinants of MS. Although many wide-spread environmental changes are known to be taking place (e.g., increasing atmospheric concentrations of CO_2_, CH_4_, and other pollutants; increasing global temperatures; a depletion of stratospheric ozone; a greater dietary consumption of trans-fats, etc.), one recent change (potentially relevant to the possible role of vitamin D deficiency) is that people are increasingly encouraged to avoid prolonged sun-exposure and to use sun-block to prevent skin cancer [87]. Nevertheless, sun-block with sun-protective-factor (SPF)-15 blocks approximately 94% of the incoming UVB radiation and higher SPF levels block even more [87]. As a result, any wide-spread use of sun-block and sun-avoidance will exacerbate any population deficiency of vitamin D synthesis and, presumably, will increase the likelihood of diseases related to vitamin D deficiency. By contrast, the pattern of EBV infection seems to have changed little over this interval [88].

## Results and Methods

### The Causal Model

**Figure 1:**
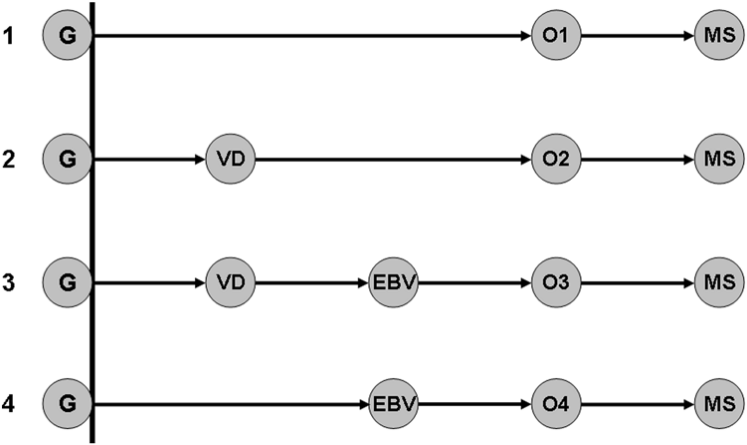
Possible causal pathways (1–4) leading to MS, which include genetic factors (G), vitamin D deficiency (VD), Epstein-Barr virus (EBV) infection, and other unidentified environmental factors (O1–O4). The other environmental factors along the different pathways to MS have been designated O1–O4 because these relevant (but unknown) factors need not be the same along each path. In the Figure, the first two environmental events are represented as an appropriately‐timed vitamin D deficiency and an appropriately‐timed EBV infection. However, the association of these specific events with the first and second environmental events (discussed in the text) is not a necessary part of the Model itself.

Thus, there seems to be clear epidemiological evidence for at least three distinct environmental events contributing to MS pathogenesis. Consequently, it is unnecessary to choose *between* the vitamin D and the EBV hypotheses. They may both be correct. Nevertheless, even if these two environmental events are part of *a* pathway to adult MS, they may not be on the *same* or the *only* pathway. Indeed, assuming that each is part of *some* causal pathway, there are several possible arrangements for how these environmental-events might produce MS (Figure 1). No pathway can be excluded entirely although, if prior EBV infection is necessary for adult MS (Table 1), then pathways 1 and 2 (Figure 1) must occur rarely, if at all. Similarly, if pathway 4 were the major pathway, an observable maternal effect would not be anticipated. Consequently, only pathway 3 (implying sequential environmental events) seems to form a necessary part of causal cascade to adult MS. The first two events may be an appropriately-timed vitamin D deficiency and an appropriately-timed EBV infection (as in the Figure). However, these particular associations with the first and second environmental events are not a necessary component of the Model and, if it turns out that these two factors are not relevant, then two suitable alternatives would simply need to be substituted into the equations without any alteration of the Model itself.

Definitions for the terms used in the Model are presented in Table 2. The life-time probability of getting MS (P_MS_) will be equated with the prevalence of MS in the general population (Table 2). This is an approximation, which is accurate only if disease incidence is unchanging, mortality is unaffected, the population size is stable, and the diagnostic sensitivity (over the entire life-span) is unchanged. None of these conditions pertain exactly. Nevertheless, this assumption seems reasonable as a rough approximation, especially because the error introduced by increased mortality (in the estimate for individuals currently aged 35–40 years) will be opposite in direction from the others. The probability of genetic susceptibility (P_G_) will refer to the probability of an individual possessing any of possibly several “susceptible” genotypes and will refer to the susceptibility conferred by genetic determinants present at conception. Alterations of genetic expression subsequently will be considered environmental events although the genotypes that permit such alterations to occur would still be included within the group of “susceptible” genotypes. Because mitochondrial genes linked to MS have not been found [2] and because, as discussed above, preferential maternal transmission has not been observed [19], [20], genetic susceptibility will be attributed to nuclear genes.

**Table 2:** Definition of Terms used in the Model.

Time Period 1	=	the period of (1941–1945)*
Time-Period 2	=	the period of (1976–1980)*
R	=	Percentage of women in the MS population (i.e. if the ratio of women to men in the population is 3.2∶1, then: R = (3.2)/(4.2) = 0.76).
P_G_	=	the probability (i.e., the prevalence) of genetic susceptibility (gender not specified)
	P_GW_	= the probability of genetic susceptibility in women
	P_GM_	= the probability of genetic susceptibility in men
P_E_	=	probability that a “sufficient” environmental exposure occurs (all factors; gender not specified)
	P_EW_	= probability that a “sufficient” environmental exposure occurs (all factors; in women)
	P_EM_	= probability that a “sufficient” environmental exposure occurs (all factors; in men)
P_G,E_	=	the probability that both genetic and environmental exposures occur (gender not specified)
CR_MZ_	=	the proband-wise monozygotic-twin concordance-rate (gender not specified)
	Zm	= the proband-wise concordance-rate (CR_MZ_) in men
	Zw	= the proband-wise concordance-rate (CR_MZ_) in women
P_E_*	=	(P_E_|G)(P_MS_|G, E) = CR_MZ_
	=	the probability of an “effective” exposure in a “susceptible” individual, including both the necessary environmental and random events (gender not specified)
	P_EW_*	= (P_EW_|G_W_)(P_MS_|G_W_, E_W_) = Zw
		= the probability of an “effective” exposure in a “susceptible” individual, including both the necessary environmental and random events in women
	P_EM_*	= (P_EM_|G_M_)(P_MS_|G_M_, E_M_) = Zm
		= the probability of an “effective” exposure in a “susceptible” individual, including both the necessary environmental and random events in men
P_VD_	=	probability of a “sufficient” vitamin D deficiency at an appropriate time
P_EBV_	=	probability of a “sufficient” EBV infection at an appropriate time
P_O_	=	probability of a “sufficient” exposure to other unidentified factors at an appropriate time
P_MS_	=	the probability (prevalence) of MS in the population (expressed in cases per 100,000 population; gender not specified).
C	=	(P_MS1_) / (P_MS2_)
	=	the ratio of the previous to the current prevalence (probability) of MS in the population; (C≥0).
**r**	=	the hazard-rate for “effective” exposure to environmental factors
**x**	=	the level of actual environmental exposure experienced by the population.
**λ**	=	**λ_w_**−**λ_m_**
	=	the difference in threshold level of actual environmental exposure between men (i.e., when: **rx**+**λ_m_** = 0) and women ((i.e., when: **rx**+**λ_w_** = 0). This threshold is the level of exposure below which disease is not possible. (NB: **λ_w_** = **λ_m_**+**λ**)
**X**	=	**r · x**
	=	the product of the actual level of exposure and the hazard-rate for “effective” exposure to environmental factors. One exposure unit (whatever this represents) is defined such that: **rx**_2_ = **rx**_1_+1. (i.e., the exposure unit is scaled such that: X_2_−X_1_ = 1)

* Subscripts are used to designate the level of different parameters at different time-periods [e.g., X_1_, Zw_1_, Zm_1_, R_1_, P_EW1_*, P_EM1_*, and P_MS1_ are the levels of these parameters in (1941–1945); and X_2_, Zw_2_, Zm_2_, R_2_, P_EW2_*, P_EM2_*, and P_MS2_ are the levels of these parameters in (1976–1980)].

Initially, the probability (P_E_) will be used to describe the combined occurrence of an entire set of environmental events (taking place at appropriate times) that could cause MS in, at least, some genetic backgrounds. Such an occurrence will be referred to as an individual experiencing a “sufficient” set of environmental events. Here, it is understood that it is possible that every “sufficient” set of environmental events may not be “sufficient” for every “susceptible” genotype. However, the set of environmental events does need to be “sufficient” for, at least, one such genotype. Under these conditions, the probability that any individual will develop MS is described by the equation:
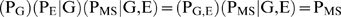
(1)where (P_E_|G) is the conditional probability of an individual experiencing a “sufficient” set of environmental events given that they are genetically susceptible to MS. The last term (P_MS_|G, E) is the conditional probability of MS developing in an individual having both an appropriate genetic make-up and experiencing a “sufficient” set of environmental-events. As such, this last term allows for different probabilities of developing MS in persons with different combinations of “susceptible” genotypes and “sufficient” environmental exposures. It also allows for a purely stochastic factor, in which only a fraction of individuals will actually develop MS, even when they are genetically susceptible and when they experience an environmental exposure “sufficient” for MS to develop given their particular genotype. Importantly, however, Equation (1) is neither speculative nor controversial and it does not limit the environmental or genetic possibilities. It is simply the definitional statement for these various conditional probabilities.

Nevertheless, although it is clear that genetic susceptibility plays a key role in MS pathogenesis, theoretically, it could also turn out that everyone in the population might become susceptible to MS in response to some very special environmental circumstances, regardless of their genetic make-up. In this case, Equation (1) would require some modification (see Appendix S1). Despite this possibility, however, the available experimental evidence suggests that the large majority (perhaps all) of MS is due to the effect of specific environmental events acting on genetically susceptible individuals and, thus, that Equation (1) adequately characterizes the causal pathway leading to MS (see Appendix S1 for an expanded discussion of these issues).

For a monozygotic-twin of an MS proband, (P_G_) should be close to 100%. Even though gene copy numbers can vary and epigenetic factors may differ to a degree between monozygotic-twins, especially as the individuals age [89], [90], in general, any monozygotic-twin of an MS proband will be genetically susceptible if this is conferred solely by nuclear genes inherited at conception. Moreover, as discussed in Appendix S1, for a monozygotic-twin of an MS proband, the lifetime probability of developing MS (P_MS_) is equal to the proband-wise concordance-rate of MS (CR_MZ_). Thus, in this special case, where (P_G_≈1) and where (P_MS_≈CR_MZ_), Equation (1) simplifies to:
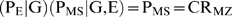
(2)


Thus, the product of the conditional probability of experiencing a “sufficient” environmental exposure and the probability of an appropriate outcome from a random (stochastic) process is equal to the proband-wise monozygotic-twin concordance-rate (which is also equal to the penetrance of the complex genetic trait). This relationship is independent of whether any specific factor is in the causal path to MS and the value of [(P_E_|G)(P_MS_|G, E)] can be determined regardless of whether genetic susceptibility, environmental exposure, and any stochastic processes are independent of each other. As a result, if both (P_MS_) and (CR_MZ_) are known for any particular region, the prevalence (probability) of genetic susceptibility (P_G_) in the population for that region can always be approximated as:
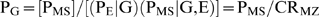
(3)


Note that in deriving Equation (3), beyond ascribing susceptibility to nuclear genes, no assumptions have been made with respect to the environmental events, the genetic variables or their interactions. As noted, Equation (1) follows directly from the definition of conditional probability and Equations (2) and (3) are immediate consequences of this. Moreover, each of the potential errors (discussed earlier), which arise from equating (P_MS_) with the prevalence of MS in the general population, will also arise from equating (CR_MZ_) with the prevalence of MS in monozygotic-twins of an MS proband. Therefore, in Equation (3), these errors (whatever they are) should largely cancel.

As an example, for far northern populations [9]–[11], the range of estimates for (CR_MZ_) is 20–30% and for (P_MS_) is 0.1–0.2%. Therefore, (P_G_) in these regions can be approximated as 0.3–1.0% (Table 3). In more southerly regions of North America and Europe, both rates are approximately half those in the north [11]–[13], implying that (P_G_) is in the same range (Table 3). In fact, applying Equation (3) to every region in which (P_G_) can be calculated from the available epidemiological data, (P_G_) seems to be in a very similar range everywhere [9]–[13], [91], [92], without any obvious north-south gradient (Table 3). It may be (as is often suggested) that the probability of genetic susceptibility is different in some ethnic populations than in others although, at present, based on the apparent absence of any difference in susceptibility between the ethnically different populations of Europe and North America (Table 3), such a possibility is pure speculation. Thus, all that can be said at the moment is that throughout Europe and North America, the probability of being genetically susceptible is remarkably consistent (Table 3).

**Table 3:** Prevalence (probability) of genetic susceptibility in populations in different geographic regions.

Location	MS Prevalence‡ (P_MS_)	MZ Concordance* (CR_MZ_)	Latitude	% Susceptible** (P_G_)
**North America**				
Canada [10]	68–248	25.3%	N45–60°	0.3–1.0
Canadian Women***	152.4	34.0%		0.45
Canadian Men***	47.6	6.5%		0.73
Northern US [11]	100–160	31.4%	N41–45°	0.3–0.5
Southern US [11]	22–112	17.4%	N30–41°	0.1–0.6
**Europe**				
Finland [91]	52–93	46.2%	N60–70°	0.1–0.2
Denmark [92]	110	24%	N55–58°	0.5
British Isles [9]	74–193	40.0%	N50–59°	0.2–0.5
France [12]	32–65	11.1%	N44–50°	0.3–0.6
Sardinia [13]	144–152	22.2%	N39–41°	0.6–0.7
Italy [13]	38–90	14.5%	N38–46°	0.3–0.6

‡ Per 100,000 population. The prevalence of MS (i.e., the measure used to estimate P_MS_) for each region is taken from data provided in [51]. A range is given because, often, a range of estimates are available for a particular region.

* Studies (9,11, and 12) reported pair-wise monozygotic-twin (MZ) concordance-rates and these have been converted into proband-wise rates assuming a random sampling of twin-pairs (see Appendix S1). Also, the error associated with the estimate of CR_MZ_ for each region has not been taken into account.

** Calculated according to Equation (3):
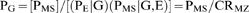
See text for details. Because the prevalence of possessing at least one copy of the HLA DRB1*1501 susceptibility allele is 30% in the general populations of northern Europe and northern North America(i.e., P_HLA+_) and 55% in the MS populations(i.e., P_HLA+ MS_) from these regions [2], [103] and assuming approximately equal penetrance for the different susceptibility genotypes [10], the observation that (P_G_≈0.5%) indicates that only about 1% of individuals who carry this allele are even susceptible to MS. Thus: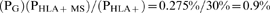.

*** Because both men and women come from the same Canadian population, the actual disease prevalence is irrelevant and, therefore, a range of estimates is unnecessary. Nevertheless, the current prevalence of MS in Canada (for the purpose of these calculations) was taken to be 100 per 100,000 population [10] and divided according to the current sex ratio of 3.2∶1 in Canada [65].

Nevertheless, it is clear that a person's genetic make-up is, by far, the most important determinant of MS risk, despite the fact that the contribution of individual genes to that risk seems to be small and that several genome-wide screens have not provided evidence for strong associations other than at the HLA DRB1 locus [2]. Thus, by this probabilistic analysis, over 99% of individuals seem to be genetically incapable of getting MS, regardless of what environmental events they experience during their lives. Paradoxically, however, because the (P_G_) term is so similar in different areas (Table 3), it is the environmental events (i.e., the P_E_ term) that determine the observed regional variations in MS epidemiology. In addition, the mechanisms underlying genetic susceptibility to MS are likely to be quite complex. Thus, even though the HLA DRB1*1501 allele has the largest and most consistent association with MS susceptibility of any genetic marker identified to date [2], approximately 99% of the individuals who carry this susceptibility allele are not even genetically susceptible to getting MS (Table 3).

For the purposes of this Model, only two time-periods from the Canadian study will be considered in detail although, in fact, the observed change in sex-ratio seems steady and consistent over the entire study-interval [65] and the parameter estimates derived from each these different sex-ratio data points (see Appendix S1) is quite similar (Table 4). The first interval considered is (1941–1945) because, of the older data points, this is the oldest with a very narrow confidence interval [65]. The second is (1976–1980), which will be considered “current” because this is the youngest 5-year cohort whose members have lived long enough (i.e., 35–40 years) for MS to declare itself. Also, this time-period is the most likely to match-up with “current” estimates of the monozygotic-twin concordance-rates. Obviously, these point estimates, are associated with error terms [10], [65], so that estimates derived from them are only valid within the limits set by these potential errors. Nevertheless, knowing that the sex-ratio in the (1976–1980) time-frame is 3.2 [65] and using an estimate of 0.1% for MS prevalence in Canada [2], [51], a gender-specific MS prevalence of 152.4 and 47.6 per 100,000 population can be calculated for women and men respectively (Table 3). Moreover, the proband-wise monozygotic-twin concordance-rate for women (0.34) is significantly larger (p<0.001) than the same rate (0.065) for men [10].

**Table 4:** Parameter estimates using different the sex-ratios (Female∶Male) reported in Canada over the period from 1931 to 1980*.

Time-Period	a	b	b/a	λ	C_max_ **
1931–1935	0.083	0.477	5.73	−0.270	0.76
1936–1940	0.079	0.465	5.86	−0.396	0.73
1941–1945	0.078	0.449	5.75	−0.373	0.76
1946–1950	0.075	0.434	5.77	−0.462	0.75
1951–1955	0.074	0.417	5.60	−0.377	0.80
1956–1960	0.072	0.403	5.58	−0.448	0.79
1961–1965	0.072	0.385	5.37	−0.231	0.89
1966–1970	0.070	0.370	5.31	−0.188	0.92
1971–1975	0.067	0.356	5.37	−0.772	0.75
**Mean** (sd)	**0.074** (0.005)	**0.417** (0.042)	**5.59** (0.2)	**−0.391** (0.17)	**0.79** (0.06)

* Values derived from reference [65] for the condition where (C = 0.5) and assuming the change in the environmental factor (whatever this is) has been steadily increasing (i.e., linear) over the time-interval (sd = standard deviation). The estimates for the parameters **a** and **b** trend downward in the more recent time-intervals because the definition of a unit of environmental change (see Table 2) becomes smaller with more recent observation periods. The other estimates are unaffected by this circumstance (see Equations 14, 17, and 21).

** Values for the maximum value that C could take (see text); extrapolated backward to the (1941–1945) time-period for comparison.

From Equation (3), and as shown in Table 3, men seem to be 60% more likely to be genetically susceptible to getting MS than are women (i.e., P_GM_>1.6 P_GW_). This result is independent of the actual prevalence of MS in Canada and, consequently, the greater current MS prevalence in women, presumably, is due to the fact that the [(P_E_|G)(P_MS_|G, E)] term in Equation (1) is presently larger for women than for men.

This gender-specific environmental effect might reflect a true difference in exposure (e.g., maybe women use more sun-block or sun-avoidance than men, maybe they spend less time out of doors, or maybe they have better hygiene as children and therefore acquire EBV later). It could also reflect gender-specific differences in vitamin D metabolism, which causes men and women to experience a deficiency at different absolute exposure levels [53], [54]. It may also reflect women having a greater probability of actually developing MS once the necessary environmental and genetic events have come together or it could be that some combination of these factors contributes to the observed gender-specific differences.

Regardless of the explanation, however, the existence gender-specific differences necessitates that Equation (1) be re-written separately for both women and men as:
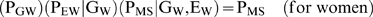
and
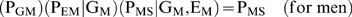



In the Model, these gender-specific terms [(P_EW_|G_W_)(P_MS_|G_W_, E_W_) and (P_EM_|G_M_)(P_MS_|G_M_, E_M_)], will be referred to (collectively) as the probability of an “effective” exposure (i.e., an exposure that actually produces disease in a susceptible individual). These gender differences also necessitate the use of gender-specific monozygotic-twin concordance-rates (Zw and Zm), as defined in Table 2.

MS epidemiology in Canada has been changing in the 35 year interval between (1941–1945) and (1976–1980). First, MS prevalence may have doubled (in which case C would be equal to 0.5) and the sex-ratio of women-to-men has increased from 2.2 to 3.2 [2], [51], [65]. From these two pieces of information, and assuming both that P_GW_ and P_GM_ are unchanged and that the current MS prevalence is 0.1%, it follows that the gender-specific MS prevalence in (1941–1945) was 68.8 and 31.2 per 100,000 population for women and men respectively and that the gender-specific term for the probability of “effective” exposure during this period (Table 2) is (0.307*C = 15.3%) for women and (0.085*C = 4.3%) for men (see Appendix S1). Moreover, knowing these values permits the relationship between the probability of an “effective” exposure and the actual exposure level of the population to be defined rather precisely (see Appendix S1).

If the hazard-rate for “effective” exposure is constant and the same for men and women, which seems plausible for a stochastic process related only to the actual exposure level received by the population (see Appendix S1 for a discussion of these issues), the most general equations describing the approach to maximum probability of “effective” exposure are exponential and are given by:
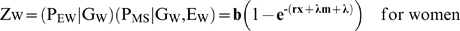
and
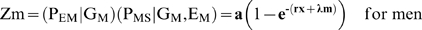



In these equations, **a** and **b** are positive constants (≤1), which represent the maximum probability of “effective” exposure for men and women, **x** (≥0) represents the actual exposure level received by the population, and **r** (≥0) is the hazard-rate for “effective” exposure. The threshold exposure level at which disease becomes possible and is defined as the exposure-level (**rx**) such that:
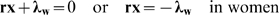


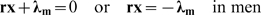



The parameter (**λ**) represents the difference (between men and women) in this threshold exposure-level, so that:
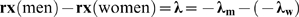


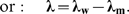



**Figure 2:**
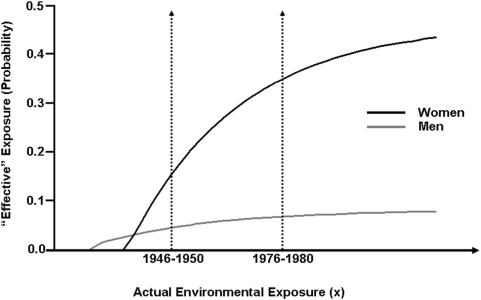
Derived response curves for men and women of an “effective” exposure to the environmental factors (P_E_*) with increasing levels of actual exposure (x), as described in the text and in the Appendix. These curves are based upon the “current” proband-wise monozygotic-twin concordance-rates for men and women in Canada [10] and upon the change in the sex-ratio observed in Canada between the two time-periods [65]. However, in deriving this set of curves, two further assumptions have been made. First it has been assumed that the currently observed values for Zw_2_ and Zm_2_ are accurate [10]. Second, it is assumed that C (defined in Table 2) is equal to one half (i.e., that the prevalence of MS has doubled between the two time intervals). Of course, the actual level of environmental exposure (whatever this represents) is unknown. Nevertheless, the environmental exposure has been scaled such that the difference in the actual exposure level between these two time-periods (whatever this is), multiplied by the unknown hazard rate, is assigned the arbitrary value of one exposure unit (see Table 2). In these curves, the probability of a “sufficient” exposure to the entire set of environmental events (P_E_) is assumed to have changed (in some manner) as the actual level of exposure (x) of the population has increased between the two time intervals of study. This change in (P_E_), however, could be due to a change in only one, in some, or in all of the relevant environmental factors. NB: Although the two time-periods of (1941–1945) and (1976–1980), which were used for parameter estimation, are shown along the x-axis, this axis represents an increasing (but unknown) environmental exposure. It is not a time-axis.

By virtue of a few basic epidemiological observations [10], [65], we can specify two points on these gender-specific response curves and, therefore, each curve can be defined within narrow limits (see Appendix S1). Thus, based upon the current proband-wise monozygotic-twin concordance-rates [10], allowing for an accuracy (±1 SE), and based upon the observed changes in sex-ratio [65], it can be shown that (**b**>**a**), that (0.018<**a**<0.154), that (0.335<**b**<0.576), that (2.7<**b/a**<26.1), and that (**λ**<−0.1). It is therefore apparent that women are more responsive to the environmental changes that have taken place (whatever they are) than men. Despite this, however, men have a lower threshold of actual environmental exposure for the disease to develop than women. Such a circumstance might explain why the earliest reports of MS were often in men [2], [93] and why a 1922 study reported that of 363 MS patients from the United States and of 1,142 cases from Europe, approximately 58% (in both regions separately) were men [94]. Moreover, it can be shown that, at a minimum, there must have been a 32% increase in the prevalence of MS in Canada over the 35 year interval of study. Some increase in MS prevalence might be expected from better diagnostic techniques although, because this minimum increase in MS prevalence depends only upon the observed change in the sex-ratio (see Appendix S1), this explanation seems unlikely. Indeed, assuming that MS prevalence has doubled over the 35 year interval [2], [51], and that the “current” estimates for Zw_2_, Zm_2_, R_2_, and R_1_ are accurate, every parameter can be determined precisely [i.e., **b** = 0.449, **a** = 0.078, **b/a** = 5.751, and **λ** = −0.373] and the theoretical response curves constructed exactly (Figure 2). Finally, these parameter estimates are quite stable for all of the changes in sex-ratio that have been observed over time in Canada (Table 4).

Thus, there can be no doubt that the environmental factors have been changing over the past several decades and probably for much longer. Nevertheless, it is possible that only some of these implicated environmental factors have changed and, thus, only these particular factors may be responsible for the changes that have taken place in MS epidemiology over the past several decades. If, as discussed earlier, the sequential pathway 3 (Figure 1) plays the dominant role in adult MS pathogenesis, then it follows that the “total” environmental term (P_E_) can be re-written as:
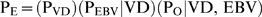
(4)


In this equation, (P_VD_) is the probability of a “sufficient” exposure to vitamin D deficiency, the term (P_EBV_|VD) is the conditional probability of a “sufficient” EBV exposure given a “sufficient” vitamin D exposure, and (P_O_|VD, EBV) is the conditional probability of the other “sufficient” environmental exposures given the fact that the individual has already experienced “sufficient” vitamin D and EBV exposures.

If individuals are equally likely to receive a “sufficient” exposure to each of these three environmental events (VD, EBV, and Other), if (P_MS_|G, E) is 100%, and if the conditional probabilities are approximately equal to the probabilities themselves, then it follows that 63% of northern European and northern North American populations [9], [10] have experienced what would have been a “sufficient” exposure to each of the three implicated environmental events in a genetically susceptible individual. Thus, in this circumstance:
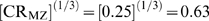



Even in the more southerly regions of these continents [11], a “sufficient” exposure to each of the events would still be experienced by 53% of the population. It is only because genetic susceptibility is so infrequent that the disease is uncommon. Moreover, if (P_MS_|G, E) is less than 100% (as seems likely), these numbers will only increase further as this probability declines. Consequently, the necessary environmental exposures in the causal pathway to MS seem likely to be very common events. Indeed, because both vitamin D deficiency and EBV infection are both very common population-wide events, this conclusion is fully consistent with these factors being the first two environmental events involved in MS pathogenesis. Thus, vitamin D deficiency (at least to some degree) is anticipated in the large majority of individuals living in low sun-exposure regions [95] and EBV is an extremely prevalent pathogen in human populations (Table 1).

## Discussion

If, as suggested by the above Model, the causal pathway leading to adult MS involves sequential environmental events or factors (pathway 3; Figure 1) then, theoretically, modification or disruption of any one of these factors has the potential to completely prevent the disease [1]. The simplest factor to modify in this way would be vitamin D deficiency because vitamin D can be supplemented easily and cheaply. However, due to the low incidence of MS, any test of such a therapeutic strategy will be very difficult to undertake. First, it will require following thousands of individuals for many (>30) years. Second, the cost of using a randomized, placebo-controlled design in this setting is prohibitive, even disregarding the ethical and logistical problems of using a placebo for such a prolonged period. And finally, any study designed to enrich the study-cohort for high-risk individuals by including only first-degree relatives of MS probands [37] will fail if the critical time for environmental exposure occurs *in utero*, during the early post-natal period, or even during childhood. Thus, by the time that MS probands are identified, most of their brothers and sisters will have already passed their window of therapeutic opportunity.

Therefore, in order to test this hypothesis, it will require the use of an open-label, observational study design that includes all women who want to become pregnant. Moreover, it will need to employ statistical methods for minimizing bias in the analysis of non-randomized data [e.g.], [ 96]–[99]. Adequate vitamin D supplementation will need to be recommended both before and during pregnancy for the mother and thereafter for both the mother and child. However, because the amount of supplementation necessary to achieve adequate blood-levels of vitamin D (i.e., in the normal range) may be quite high, it should be anticipated that some (perhaps many) obstetricians or pediatricians will be unwilling to recommend such large dosages of vitamin D either to their pregnant or to their pediatric patients. Such a circumstance, however, does not detract from the study design. In fact, a large variation in physician and patient behavior (with respect to the use and magnitude of vitamin D supplementation) will actually make the final data analysis easier [96]–[99]. As a result, in order to conduct such a trial, there is no need to encourage reluctant physicians to recommend supplementation, especially when large numbers of MS patients around the world are already consuming large quantities of oral vitamin D on a daily basis.

The current FDA recommendations suggest that 400 international units (IU) of vitamin D per day is an adequate amount of supplementation, although this recommendation is derived mostly from the effects of vitamin D on calcium homeostasis. The requirements for immune modulation, however, are likely to be higher [100]. For example, healthy men have been estimated to use between 3,000 and 5,000 IU/day [101]. Moreover, even with calcium supplementation, doses of vitamin D between 7,000 and 40,000 IU/d seem to be safe and unaccompanied by toxicity, including elevations in serum calcium concentrations or in the calcium-to-creatinine ratios [101], [102]. Consequently, dosages of between 1,000 and 10,000 IU/d day would not seem unreasonable or unsafe.

However, if physicians do recommend supplementation (at whatever level), they should monitor their patients' blood-levels to ensure that these don't exceed the range of normal. Importantly, however, the hypothesis that early vitamin D supplementation influences the subsequent likelihood of developing MS can be tested solely by acquiring observational data and does not require anyone to agree to any specific treatment plan. Nevertheless, as mentioned earlier, the conduct of such a study will require patients and their children to be followed prospectively for long periods of time – a design that, realistically, is only feasible in situations where large population-based centralized medical records are available (e.g., countries with universal health care) or in non-mobile communities where complete ascertainment and longitudinal follow-up is possible. Physicians (and their patients) could be informed about the nature of the hypothesis being tested (possibly through the MS societies or other outlets) and about the ranges of vitamin D dosages being considered (including current FDA guidelines). Such a study would be cost-effective (only information already available needs to be captured), it would be easy to accomplish (everyone can participate and can take whatever supplementation they choose), and it would pose no ethical dilemmas (each person and each physician is free to choose what they feel is best for themselves or for their patients). Moreover, this seems like an extremely important study to begin, especially in health care systems where persons and their offspring can be easily tracked. Certainly, such a study seems to carry minimal risk, it requires little cost, and, if successful, it would provide inestimable benefit for future patients. However, because it will take up to 30 years to arrive at an answer, the prospective acquisition of this kind of data should begin now.

## Supporting Information

Appendix S1 (0.14 MB DOC)
