# Supplementary material for: The Causal Cascade to Multiple Sclerosis: A Model for MS Pathogenesis
Source: PLoS One. 2009 Feb 26;4(2):e4565. doi: 10.1371/journal.pone.0004565 (PMC2644781; doi:10.1371/journal.pone.0004565)
Supplement: Appendix S1 — (0.14 MB DOC) [file pone.0004565.s001.doc]

**Appendix S1**

For clarity of the Appendix, the first four equations from the paper, which follow directly from the definitions of conditional probability, will be restated in brief:

(P_G_)(P_E_│G)(P_MS_│G, E) = (P_G,E_)(P_MS_│G, E) = P_MS_ (1)

The definitions of terms are provided in Table 2. In the special circumstance of a monozygotic-twin of an MS proband, (P_G_ ≈ 1) and (P_MS_ ≈ CR_MZ_) so that, in this special case, Equation (1) simplifies to:

(P_E_│G)(P_MS_│G, E) = (P_MS_) = CR_MZ_ (2)

As a result, if both (P_MS_) and (CR_MZ_) are known for any particular region, the prevalence (probability) of genetic susceptibility (P_G_) in the population for that region can be estimated as:

P_G_ = [P_MS_] **/** [(P_E_│G)(P_MS_│G, E)] = P_MS_ / CR_MZ_ (3)

Lastly, if there are at least three sequential environmental components in the causal chain leading to MS (assuming the relevant environmental events are vitamin D deficiency, EBV exposure, and other), then:

P_E_ = (P_VD_)(P_EBV_ │VD) (P_O_ │VD, EBV) (4)

Naturally, if vitamin D and EBV exposure are not the relevant environmental events, Equation (4) would need to be modified to include the three or more environmental events that were relevant. Because men seem to be 60% more likely to be genetically susceptible to MS than women (Table 3), the greater prevalence of MS in women (at the moment) must be due to the fact that the [(P_E_│G) (P_MS_│G, E)] term in Equation (1) is currently larger for women than for men. Such a circumstance could be due to a true difference in exposure between men and women (at any given population level of exposure), it could be due to men and women requiring different amounts of environmental exposure and, therefore, experiencing “sufficient” exposures at different levels of actual exposure for the population. It could also be due to men and women responding in a different physiological manner (e.g., 53, 54) to changing levels of actual environmental exposure, to women having a greater probability of developing MS once the necessary environmental and genetic events have come together, or it could be due to some combination of these factors. Regardless of the explanation, however, Equation (1) needs to be written separately for women and men as:

(P_GW_)(P_EW_│G_W_)(P_MS_│G_W_, E_W_) = P_MS_ (for women)

and

(P_GM_)(P_EM_│G_M_)(P_MS_│G_M_, E_M_) = P_MS_ (for men)

For the purposes of the Model, the expressions [(P_EW_│G_W_) (P_MS_│G_W_, E_W_) and (P_EM_│G_M_) (P_MS_│G_M_, E_M_)] in the above equations, collectively, will be referred to as the probability of an “effective” exposure in women and men respectively. Thus, the term “effective” exposure takes into account both the probability that an individual experiences a “sufficient” exposure (at a particular level of actual population exposure and appropriate for their genotype) and also the chance that that a genetically susceptible individual will actually develop the disease once they have experienced the necessary environmental events.

Because it seems unlikely that the genetics of MS in Canada can have changed substantially between time-periods 1 and 2 (i.e., 35 years, or 1-2 generations), the (P_G_), (P_GW_), and (P_GM_) terms will be assumed to have remained constant over this interval. In this case the constant (C), will reflect only a change in the “effective” exposure term [(P_E_*) = (P_E_│G)(P_MS_│G,E)] of Equation (1). Because the sex ratio has changed from 2.2 in the (1941-1945) time-period to 3.2 in the (1976-1980) time-period, and substituting the expressions (P_EW_*) and (P_EM_*) for (P_E_*) to distinguish and separate the effect for women from that for men, this implies that:

P_EW1_* = Zw_1_ = (R_1_)C(P_MS2_) **/** (P_GW_) = (R_1_/R_2_)C(Zw_2_) (5)

and that: P_EM1_* = Zm_1_ = (1–R_1_)C(P_MS2_) **/** (P_GM_) = [(1–R_1_)/(1–R_2_)]C(Zm_2_) (6)

**Purely Environmental Factors in MS Pathogenesis**

Despite the fact that genetic susceptibility clearly plays a key role in MS pathogenesis (2-8), it could also be the case that, in some very special environmental circumstances (e.g., E**), everyone becomes susceptible, regardless of their genetic make-up. In this case, we will let (P_MSG_) and (P_MSE_) represent, respectively, the probability of genetically-determined MS and of purely environmental MS in the general population. Thus:

(P_G_)(P_E_│G)(P_MSG_│G, E) = P_MSG_ (a)

(P_E**_)(P_MSE_│E**) = P_MSE_

and Equation (1) would need to be re-written as:

P_MSE_  + P_MSG_ – (P_MSE_)(P_MSG_) = P_MS_

Because both P_MSE_ and P_MSG_ must be less than or equal to P_MS_ (i.e., 0.1-0.2% in the general populations of northern North America and northern Europe), the cross-product term is negligible in comparison with the other two terms and can be ignored. If we let (p) represent the proportion of patients in the general population with genetically-determined MS, then:

P_MSG_ = (p)(P_MS_) (b)

and: P_MSE_ = (1– p)(P_MS_)

and, if (p = 1): (P_G_)(P_E_│G)(P_MSG_│G, E) = P_MS_ (c)

Because (P_MSE_ < P_MS_), this means that the maximum probability that an individual will experience an “effective” exposure to any purely environmental factor (E**) is very small (< 0.2%). In the case of a monozygotic-twin of an MS proband, the (P_MSE_) term will remain unchanged so that Equation (2) becomes:

P_MSE_ + P_MSG_ – (P_MSE_)( P_MSG_) = CR_MZ_

and substituting the values of (P_MSE_ < 0.002) and (CR_MZ_ = 0.25) into this equation yields:

P_MSG_ > 0.994(CR_MZ_)

so that: P_MSG_ ≈ CR_MZ_ (d)

Thus, essentially all (>99%) of the concordant-twin cases of MS probands are the result of genetic susceptibility. For the purpose of this analysis we will let (P_MS_*) represent the probability of genetically-determined MS when (P_G_ = 1) and (p = 1). In this situation, by Equation (c):

(P_E_│G)(P_MSG_│G, E) = P_MS_* (e)

and by Equations (b), (c), and (d):

: P_MSG_ = (p)(P_MS_*) = CR_MZ_

so that: (P_E_│G)(P_MSG_│G, E) = CR_MZ_ / p (f)

Also, by Equations (a), (b), and (f), Equation (3) becomes:

P_G_ = (p^2^)(P_MS_ **/** CR_MZ_)

Thus, under these conditions, the probability of an individual being genetically susceptible will be reduced from the original Equation (3) estimate by the factor of (p^2^). Because (CR_MZ_ / p ≤ 1), it follows that (0.25 ≤ p ≤ 1.0). Nevertheless, despite this wide range of theoretical possibilities for the value of (p), experimental evidence suggests that the large majority (possibly all) of the MS risk is genetically-determined (i.e., that: p ≈ 1). Thus, both using the overall probability of ascertaining an affected twin (70%) from the Canadian twin-study (10) and using the HLA-typing data reported in that study (10), it is possible to calculate the proband-wise monozygotic-twin concordance-rate (103) for probands who carry the HLA DRB1*1501 allele (CR_MZ_[HLA+]) and for probands who do not carry this allele (CR_MZ_[HLA–]). In doing these calculations it is apparent that these two rates are substantially the same (0.30 and 0.28 respectively). Moreover, the overall (i.e., combined) estimated proband-wise monozygotic-twin concordance-rate from this HLA-typed cohort is (CR_MZ_ = 0.29). The value of (p) can be estimated from the difference (Δ) between these two monozygotic-twin concordance-rates:

Δ = CR_MZ_[HLA+] – CR_MZ_[HLA–] = 0.02

Using published data in MS patients and controls from a northern Caucasian population (104), the control probability of possessing this allele is 30%, whereas this probability is increased to 55% in patients with MS (i.e., an increase of 25% above the population probability). A similar association (in both direction and magnitude) has been reported on numerous occasions in other northern European and northern North American populations (2) and clearly, therefore, this allele is implicated in the susceptibility of Caucasians to MS.

Nevertheless, because this allele is neither necessary nor sufficient for MS to develop, some MS patients who have this allele and some who don’t may be genetically susceptible for other reasons. Moreover, others who possess it may not be susceptible at all. Consequently, several possible scenarios deserve consideration in order to place these experimental findings (10) into proper context. First, it might be that HLA DRB1*1501 contributes to MS susceptibility only to the extent that this allele is over-represented in the MS population. Second, it may be that this allele contributes to susceptibility more often than this and, in the extreme case, it may always contribute. Third, the disease penetrance may differ between susceptibility genotypes that either require or don’t require this allele when conferring susceptibility. In this last circumstance, the strong *a priori* expectation (because the association between MS and this particular allele has been, by far, the easiest to identify) is that those genotypes in which this allele contributes to susceptibility will have a greater penetrance than those genotypes in which this allele does not contribute.

To analyze the implications of the first of these scenarios, we will let (x) represent the proportion of MS patients who are not susceptible due to having the HLA DRB1*1501 allele, but who are still genetically susceptible for other reasons (0 ≤ x ≤ 1); we will let (r_1_) represent the probability of an individual possessing at least one copy of the HLADRB1*1501 allele in the general population; and we will let (r_2_) represent the percent increase above the general population in the likelihood of an MS patient possessing this allele. In this circumstance:

p = [(1 – r_1_ – r_2_)(x)] + [r_2_ + (r_1_)(x)] = [r_2_ + (1 – r_2_)(x)] (g)

and, by Equation (f) and assuming equal penetrance of the different genotypes, then:

CR_MZ_(HLA+) = [1/(r_1_ + r_2_)][r_2_ + (r_1_)(x)][CR_MZ_ / p] (h)

and: CR_MZ_(HLA–) = [x][CR_MZ_ / p] (i)

so that : Δ = [r_2_/(r_1_ + r_2_)][1– x][CR_MZ_] **/** [r_2_ + (1 – r_2_)(x)]

or: x = [(r_2_/{r_1_ + r_2_})(CR_MZ_) – Δ(r_2_)] **/** [(r_2_/{r_1_ + r_2_})(CR_MZ_) + Δ(1 – r_2_)] (j)

Substituting the observed (known) values of (Δ = 0.02), (CR_MZ_ = 0.29), (r_1_ = 0.3), and (r_2_ = 0.25) into Equation (j), yields the point estimate of (x = 0.86) and, substituting this value into Equation (g) gives the point estimate for (p) of:

p = 0.90

In analyzing the implications of the second of these scenarios, Equation (j) becomes:

x = [({br_1_+ r_2_}/{r_1_+ r_2_})CR_MZ_ – Δ(br_1_+ r_2_)] **/** [({br_1_+ r_2_}/{r_1_+ r_2_})CR_MZ_ + Δ(1– br_1_– r_2_)]

where b is a constant (0 ≤ b ≤ 1). In this case, the estimated values for (x) will increase with increasing values of (b) and, therefore, the estimated value of (p) will always be greater than that from the first scenario.

In analyzing the implications of the third of these scenarios, there are two potential orders for the relationship. First, susceptible genotypes that require the HLA DRB1*1501 allele may have a greater (or equal) penetrance compared to those genotypes that do not require it (i.e., the *a priori* expectation). In this case, the estimated value for (p), as in the second scenario, will always be greater than (or equal to) the estimate from the first scenario.

By contrast, the second potential order is that susceptible genotypes, which do not require this allele, might have a greater penetrance than those genotypes, which do require it. To analyze this potential relationship, we will let (CR_MZH+_) be the proband-wise monozygotic-twin concordance-rate for those individuals in whom HLA DRB1*1501 contributes to susceptibility and we will let (CR_MZH–_) be the same rate for those in whom this allele doesn’t contribute. Moreover, we will assign (z) such that:

CR_MZH–_ = (z)(CR_MZH+_); z > 1

In this circumstance the Equations (h) and (i) for the (HLA+) and (HLA–) cohorts would become:

CR_MZ_(HLA+) = [1/(r_1_ + r_2_)][r_2_ + (r_1_)(zx)][CR_MZH+_ / p]

CR_MZ_(HLA–) = [zx][CR_MZH+_ / p]

Consequently, Equation (j) becomes:

zx = [(r_2_/{r_1_ + r_2_})(CR_MZH+_) – Δ(r_2_)] **/** [(r_2_/{r_1_ + r_2_})(CR_MZH+_) + (1 – r_2_)Δ]

giving the same point estimate of (zx = 0.86) as before. Moreover, Equation (g) can also be re-written in terms of the variable (zx) as:

p = [r_2_ + (1 – r_2_)(zx)/(z)] (k)

However, because it must be the case that:

(r_2_)(CR_MZH+_) + (1-r_2_)(zx)(CR_MZH+_) = CR_MZ_ (m)

and that: (z)(CR_MZH+_) ≤ 1.0 (n)

Therefore, substituting the estimated (known) values of (zx = 0.86), (r_2_ = 0.25), and (CR_MZ_ = 0.29) into Equations (m), (n) and, finally, (k) indicates that:

CR_MZH+_ = 0.32; z ≤ 3.1; and x ≥ 0.28

At the extreme (i.e., z = 3.1), this gives the point estimate of (p = 0.46) so that, even in this situation, genetically-determined MS would still account for almost half of MS cases. Notably, however, this extreme case requires the penetrance of susceptibility genotypes lacking the HLA DRB1*1501 allele to be 100% compared to only 32% when this allele is present. Such a marked disparity in penetrance seems highly implausible, especially when considering that the allele with the strongest and most consistent association with MS of any genetic marker (2) is the one associated with the markedly lower penetrance. Indeed, it hard to imagine that there could be any substantial penetrance imbalance favoring those genotypes, which have been considerably more difficult to discover experimentally, compared to those that have been identified easily and repeatedly (2). Moreover, as (z) decreases and approaches 1 (i.e., as this potential ordering becomes more plausible), the estimated value of (p) increases and approaches that predicted by the first scenario.

Naturally, because the original clinical observations (10) could not exclude the possibility that (Δ = 0), it is also not possible (under any of these envisioned circumstances) to exclude the possibility that (p = 1). Therefore, each of these analyses (both individually and collectively) suggests that the large majority of patients with MS (possibly all) develop this disease because of being at risk genetically and not because of any purely environmental events.

In addition, because, as indicated in Equation (d) above, the vast majority of monozygotic-twins who are concordant for MS develop the disease due (in part) to genetic susceptibility, there is another method to estimate the proportion of MS occurring in genetically susceptible individuals. For example, in Finland, which has a population-based twin-registry, a total of 3,083 monozygotic twin-pairs born prior to 1957 were reported (91). In 21 of these pairs, at least one twin had MS and, of these, 10 pairs (3 concordant for MS) agreed to participate in the study. Assuming that monozygotic twins are neither more nor less likely to get MS than the general population and non-biased sampling of the twin pairs, the prevalence of genetically determined MS in Finland can, therefore, be estimated as:

P_MSG_ = (3)(21/10)(1/3083) = 204 per 100,000 population

This is estimated prevalence of MS in monozygotic twins is considerably higher that estimated for the entire population of Finland (Table 3) and, therefore, this suggests that all MS in Finland is found in genetically susceptible individuals.

**Proband-wise Concordance-rates**

Suppose that each twin-pair can be arranged (e.g., by birth order) in such a way that the ordered pair (Mm) represents a twin-pair in which the first twin has MS (M) and the second does not (m). Excluding twin-pairs in which neither twin has MS and with random ascertainment of twin-pairs, the probabilities of the differently ordered pairs (P_MM_ + P_Mm_ + P_mM_ = 1) can be grouped into those that represent concordant twin-pairs (C = P_MM_) and those that represent discordant twin-pairs (D = P_Mm_ + P_mM_ = 2P_Mm_). In the context of the proposed Model, the probability of interest is the conditional (life-time) probability (CR_MZ_) that the second twin will develop MS given the fact that the first twin is known to have MS. This probability is:

CR_MZ_ = P_MM_ / (P_MM_ + P_Mm_) = C / (C + D/2) = 2C / (2C + D)

This is the proband-wise (or case-wise) concordance-rate (103). Naturally, the conditional (life-time) probability that the first twin will get MS given the fact that the second is known to have the disease will also be equal to the proband-wise concordance-rate. With non-random sampling (i.e., when all twin-pairs with at least one affected member do not have an equal chance of inclusion in the sample), the estimated proband-wise estimate will be reduced (103) and, in the extreme, it will be to equal the pair-wise concordance-rate [i.e., C / (C + D)].

**Standard Survival Analysis Methods**

The following four functions, taken directly from standard Survival Analysis methods (105), are useful to define:

1. F(x) = a mathematical function describing the cumulative probability that

an individual will have received an “effective” exposure to an environmental event (i.e., that they will have failed) when the population experiences a particular level (**x**) of actual environmental exposure. This is directly analogous in Survival Analysis to the cumulative probability that an individual will have died (i.e., that they will have failed) when the person has aged to a particular time (t).

2. 1- F(x) = S(x) = Cumulative probability function of survival

(by definition)

3. f(x) = d[F(x)] **/** dx = – d[S(x)] **/** dx = probability density function

for failure (by definition)

4. h(x) = f(x) **/** S(x) = hazard-rate function (by definition)

5. h(x) dx = – d[S(x)] **/** S(x) (from 3 and 4)

Computing the definite integral of both sides of the hazard-rate function (from an **x** level of exposure to a 0 level of exposure) yields:

ln [S(x)] = - **⌠^x^** h(x) dx = – **rx** ; whenever h(x) = **r** is a constant

**⌡_0_**

Taking the anti-log of both sides yields: S(x) = e^-^**^rx^** and, thus: F(x) = (1 – e^-^**^rx^**)

**The Causal Model**

In the case of a population-wide environmental exposure, the risk (i.e., the probability of any one susceptible individual receiving an “effective” exposure) seems likely to be a stochastic (random) event based only upon the actual level of environmental exposure received by the population. In this case, it seems plausible that the hazard-rate would be constant and the same in men and women (see next section for an expanded discussion of the possibility of gender-specific hazard-rates). If so, the cumulative probability of survival (i.e., of a person not having received an “effective” exposure) would be described by:

S(x) = e^-rx^  = e^-X^

where (X = rx). By contrast, the cumulative probability of failure (i.e., of a person having received an “effective” exposure) is described by:

F(x) = 1 – S(x) = (1 – e^-X^) (7)

Importantly, however, even for circumstances in which the “true” hazard function is not a constant, the use of a constant hazard for analytic purposes is still appropriate. For example, if we let f(x) be a function of x and r be a constant such that [h(x) = r ∙ f(x)], if we let F(x) be the definite integral of the function f(x) from an (x) level of exposure to a (0) level, and if we transform (x) units of exposure into (y) units such that [y = F(x)], then it follows that:

**⌠^x^** h(x) dx = **⌠^x^** r ∙ f(x) dx = **⌠^y^** r ∙ dy = **ry**

**⌡_0_ ⌡_0_ ⌡_0_**

As a result, under conditions where the hazard rate is not constant, the Model definition of an exposure unit ( ry_2_ – ry_1_ = 1), effectively transforms the exposure units from (x) to (y) and results in an “apparently” constant hazard (Table 2). Therefore, despite the fact that, under these circumstances, the abscissa no longer linearly increments the actual exposure, all of the relationships derived in the Model, as well as the response curves depicted in Figure 2, will be unchanged from those derived for a “true” constant hazard. Thus, the use of a constant hazard is always to be appropriate for the purposes of the Model.

Moreover, unlike true survival analysis (where everyone eventually dies given enough time), however, there is no theoretical reason that the probability of experiencing an “effective” exposure to an environmental event (i.e., an exposure sufficient actually to induce disease in a susceptible individual) needs to increase to 100% as the actual level of environmental exposure increases. For instance, us ing a completely hypothetical example in which vitamin D deficiency is conceived as the only necessary environmental event, this might describe a situation in which (by random chance) only 25% of genetically susceptible individuals, who experience an “sufficient” vitamin D deficiency (appropriate for their genotype) during the critical time-period, will actually develop MS. In this case, 25% would represent the limiting value for failure (i.e., “effective” exposure) in the genetically susceptible population. Also there is no theoretical reason why men and women need to approach the same limiting value for the probability of “effective” exposure. Neither does the level of exposure at which disease begins to become possible (i.e., the threshold) need to occur at any particular value of actual environmental exposure (e.g., zero), nor does this threshold need to be the same for men and women. Consequently, the most general form for Equation (7) needs to be written differently for men and women as:

(P_EW_*) = **Zw = b(1** – **e^-(X+λ^**^m^**^+λ)^ )** for women (8)

and (P_EM_*) = **Zm =** **a(1** – **e^-(X+λ^**^m^**) )** for men (9)

where **a** and **b** are positive constants (≤ 1) and represent the maximum attainable level of “effective” exposure for men and women respectively, and where it is understood that the threshold level need not be zero. The term **λ** represents the difference in threshold between women and men (i.e., **λ = λ_w_ – λ_m_**). If **a** and **b** are equal, then men and women approach the same limiting probability of “effective” exposure. If **a** and **b** are both 1.0, then (as in true survival analysis) everyone ultimately fails. If **λ** is 0, then men and women have the same threshold for the actual level of environmental exposure at which disease becomes possible. Fortunately, by virtue of a very few basic epidemiological observations made from Canada (10, 65), two points on each of these exponential curves (for that geographic region) are easily determined and, therefore, these curves can be theoretically defined within the limits set by standard errors (SE) of the measurements themselves. For example, the following four equations are immediate consequences of the terms and definitions provided (Table 2) and from Equations (5), (6), (8), and (9):

P_EW2_* = **Zw_2_** = **b(1** – **e^-(X1+1+λ^**^m^**^+λ)^ ) =** (0.762)(P_MS2_) **/** (P_GW_) **=** 0.34 (10)

P_EM2_* = **Zm_2_** = **a(1** – **e^-(X1+1+λ^**^m^**) ) =** (0.238)(P_MS2_) **/** (P_GM_) **=** 0.065 (11)

P_EW1_* = **Zw_1_** = **b(1** – **e^-(X1+λ^**^m^**^+λ)^ ) =** (0.688)(P_MS1_) **/** (P_GW_) = 0.307C (12)

P_EM1_* = **Zm_1_** = **a(1** – **e^-(X1+λ^**^m^**) ) =** (0.313)(P_MS1_) **/** (P_GM_) **=** 0.085C (13)

Naturally, the analysis of gender-specific environmental effects on adult MS pathogenesis depend upon the CR_MZ_ estimates (Table 2) for men and women (10) as being accurate for the Canadian population born in the 1976-1980 time-frame and that the reported sex ratio changes and current prevalence data (51, 65) are also accurate estimates of the underlying parameters.

If so, several conclusions are apparent from a review of these equations and from Table 3. First, from a version of Equation (3), modified for separate analysis of women and men, and as presented in Table 3, the probability (prevalence) of genetic susceptibility in men (i.e., P_GM_) is more than 60% greater than that in women (i.e., P_GW_). This result is independent of the actual prevalence of MS but does depend upon the accuracy of the monozygotic-twin concordance-rates and the sex-ratio information (10, 65). Second, from Equations (10) and (11) it is apparent that (**b** > 0.34) and (**a** > 0.065). Third, if the prevalence of MS (P_MS_) is either increasing or stable between the two time-periods, then (C ≤ 1). Fourth, although it is theoretically possible that (C ≥ 0), it seems implausible that the prevalence of MS in Canada could have more than quadrupled over the 35 year interval (2, 51). Consequently, the reality must be that (C > 0.25).

Equation (10) can be rearranged to yield: (Zw_2_ – **b**) **/** **b** = – (e^-(X1+^ **^λ^**^m^**^+λ^**))e^-1^

Similarly, Equation (12) can be rearranged to yield: (Zw_1_ – **b**) **/** **b** = – (e^-(X1+^**^λ^**^m^**^+λ^**))

And, therefore, dividing these yields: [(Zw_2_ – **b**) **/** (Zw_1_ – **b**)] = e^-1^

Similarly, rearranging Equations (11) and (13) yields: [(Zm_2_ – **a**) **/** (Zm_1_ – **a**)] = e^-1^

Therefore, putting together Equations (5), (10) and (12) with the above equation yields:

**b** = Zw_2_[1 – {R_1_/ R_2_}Ce^-1^] **/** [1 – e^-1^] (14)

or **b** = 0.538 – 0.179C (15)

[which can be re-written as: C = 3.01 – 5.60**b**] (16)

NB: for (C ≥ 0), as it must because P_MS1_ cannot be less that 0, then (**b** ≤ 0.538). If the condition of (C > 0.25) is imposed, then (**b** < 0.493).

Similarly, putting together Equations (6), (11) and (13) with the above equation yields:

**a** = Zm_2_[1 – {(1–R_1_)/(1–R_2_)}Ce^-1^] **/** [1– e^-1^] (17)

or **a** = 0.1028 – 0.0496C (18)

[which can be re-written as: C = 2.071 – 20.141**a**] (19)

NB: for (C ≥ 0), as it must because P_MS1_ cannot be less that 0, then (**a** ≤ 0.103). If the condition of (C > 0.25) is imposed, then (**a** < 0.090).

Combining Equations (16) and (19) gives:

**b** = 3.596**a** + 0.168 (20)

We can also rearrange Equations (10) (11), (12), and (13) so that:

[(Zw_2_) – **b**] = – **b**(e^-(X1+1+^ **^λ^**^m^)e**^- λ^**

[(Zm_2_) – **a**] = – **a**(e^-(X1+1+^ **^λ^**^m^))

[(Zw_1_) – **b**] = – **b**(e^-(X1+^ **^λ^**^m)^)e**^- λ^**

[(Zm_1_) – **a**] = – **a**(e^-(X1+^ **^λ^**^m^))

Combining and rearranging these, we get two equations:

[**a/b**][(Zw_2_ – **b**) / (Zm_2_ – **a**)] = e^-λ^

[**a/b**][(Zw_1_ – **b**) / (Zm_1_ – **a**)] = e^-λ^

which can then be rearranged to:

λ = – ln{[**a/b**][(Zw_2_ – **b**) **/** (Zm_2_ – **a**)] } (21)

and: λ = – ln [**a/b**][(R_1_/ R_2_)(Zw_2_)C – **b**) **/** {(1–R_1_)/(1–R_2_)}(Zm_2_)C – **a**)] (22)

In fact, knowing just one of all of the unknown parameters in the above equations (Zw_1_, Zm_1_, **λ**, **a**, **b,** C, or even the just the ratio **b/a**), the values of all the others can be determined precisely based only on the additional knowledge of the change in the sex-ratio over time and on the “current” monozygotic concordance rates for men and women (10, 65). The only assumption that is required is that the observed change in the epidemiology of the disease is not to the result of a change in the nature of genetic susceptibility to MS (which seems unlikely over such a short interval).

However, even within the limits of our current knowledge, there are certain boundaries that can be placed on the values that each of these parameters can possibly take, which can be deduced from the data we already possess. For example, because, from Equation (11), it must be the case that (**a** > Zm_2_ = 0.065) and because (C ≥ 0), then, from Equation (18), it must also be the case that (C < 0.76). That is, Canada must have experienced at least a 32% increase in the prevalence of MS between (1941-1945) and (1976-1980). It turns out that this result is independent of any possible error in the estimated rate of monozygotic-twin concordance for men. Thus, it follows from Equations (11) and (17) that:

Zm_2_ < **a** = [Zm_2_ – (Zm_1_)e^-1^] **/** [1 – e^-1^]

which can be re-written as:

[Zm_2_/(1 – e^-1^)] – Zm_2_ > [(1–R_1_)/(1–R_2_)](Zm_2_)Ce^-1^/ (1 – e^-1^)

or, equivalently:

[(1)/(1 – e^-1^) – 1] **/** [{(1–R_1_)/(1–R_2_)}e^-1^ / (1 – e^-1^)] = 0.76 > C (23)

Thus, the requirement that (C < 0.76) depends entirely upon the observed change in sex-ratio over time (65) and is independent of the correct value of Zm_2_ itself.

As a result of Equation (20), it is clear that (**b** > **a**). Moreover, from Equations (11) (18), and (20), together with the requirement (C > 0.25), it is clear that the actual ratio of (**b/a**) must be (5.4 < **b/a** < 6.2). Also, from Equations (10), (11), (15), and (18), together with (C >0.25), it follows that (0.065 < **a** < 0.090) and that (0.402 < **b** < 0.493). These conclusions are dependent upon the accuracy of the observed values (10) for (Zw_2_ = 0.34, SE = 0.057) and (Zm_2_ = 0.065; SE = 0.046). The fact that (**b > a**) depends both upon an increasing female-to-male sex-ratio and the fact that (Zw_2_ > Zm_2_). The former condition seems clear (65) and the latter condition also seems inescapable because the observed difference in the proband-wise monozygotic-twin concordance-rate between men and women in the Canada (10) is highly significant (p < 0.001).

Moreover, if we allow Zm_2_ to range from 0.018 to 0.111 and we let Zw_2_ to range from 0.28 to 0. 397 (i.e., ± 1 SE) based on the published data (10), and if we let C range from 0.25 to 0.76 (i.e., the limits for possible values of C), then we can calculate that (0.018 < **a** < 0.154), that (0.335 < **b** < 0.576), and that (2.7 < **b/a** < 26.1). If we allow Zm_2_ and Zw_2_ to range (±2 SE) under the same conditions described above, the estimates for these parameters are (0 < **a** < 0.218), (0.267 < **b** < 0.659), and (**b/a** > 1.5).

In addition, because (0 < C < 0.76), we also know from Equations (10), (11), and (21) that (**λ** < 0). This means that men have a lower threshold of actual environmental exposure than women in order to develop MS. Indeed, substituting, the permissible values of (0.25 < C < 0.76), results in (**λ** < -0.1). Perhaps this lower threshold for men, in part, explains why so many of the early reports of MS are from men (2, 91) and why some early statistics in MS actually suggested that men were more likely to develop MS than women (93). Because, by Equations (14) and (17), Zw_2_ and Zm_2_ are common factors in the both numerator and denominator of Equation (22), this equation can be rewritten as:

**λ** = -ln [[((R_1_/R_2_)C – [1 – (R_1_/R_2_)Ce^-1^] / [1 – e^-1^])

*([1 – {(1–R_1_)/(1–R_2_)}Ce^-1^] / [1 – e^-1^])]

**/ [**({(1–R_1_)/(1–R_2_)}C – [1 – {(1–R_1_) /(1–R_2_)}Ce^-1^] / [1 – e^-1^])

*([1 – (R_1_/R_2_)Ce^-1^] / [1 – e^-1^])]] (24)

This seems like a complicated expression but, as was the case for C above in Equation (23), it is provided only to demonstrate that the value of **λ** depends only upon the change in sex-ratio and disease prevalence over time (65). Thus, the fact that men have a lower threshold of actual exposure necessary for getting MS compared to women (i.e., **λ** < 0) is independent of the accuracy of the “current” estimated proband-wise monozygotic-twin concordance-rates (Zm_2_ and Zw_2_).

The potential precision of the estimates for these parameters can be exemplified by two hypothetical examples. If the prevalence of MS has doubled over the time interval (i.e., if C = 0.5), as might be suggested by the actual data (2, 51), then each of these unknown parameters in the above equations can be calculated precisely, within the errors of the original data [i.e., **b** = 0.449, **a** = 0.078, **b/a** = 5.751, and **λ** = -0.373]. This is the data that has been used to generate Figure 2. Alternatively, under the circumstances where C = 0.7, then: [**b** = 0.413, **a** = 0.068, **b/a** = 6.065, and **λ** = -1.364].

Importantly, also, the stability of these parameter estimates over time can be assessed because sex-ratio status in Canada (65) has been reported for each 5-year interval between 1931 and 1980. Consequently, the same estimating equations can be applied independently to each of these observations (Table 4). It is of note both that the sex-ratio in Canada has been steadily increasing over time (65) and that these estimates from every time-period lead to very similar estimates for each of the underlying parameters (Table 4).

**Possibility of Gender-Specific Differences in Hazard-Rate**

In the main manuscript and the Causal Model section above, the hazard-rate (r) for “effective” exposure is assumed to be both constant and the same for men and women. A constant hazard seems analytically appropriate for the Model under any circumstances (see discussion above) although the equality of the hazard-rate for men and women may not be. Therefore, in order to assess the implications of the possibility of gender-specific hazard-rates, we will let (**x_0_**) be the threshold level of environmental exposure when (Zw_0_ = 0), we will let (r_w_) be the hazard-rate for women, and we will let (r_m_) be the hazard-rate for men. Thus, from Equation (12):

[**b** – Zw_0_] / **b** = e^– (rw ∙ x0^ ^+^ **^λ^**^m+^ **^λ^**) = 1

or equivalently: r_w_∙x_0_ + λ_m_+ λ = 0 (a1)

Also by Equation (12), at an (x_1_) level of exposure yields:

[**b** – Zw_1_] / **b** = e^– (rw ∙ x1^ ^+^ **^λ^**^m+^ **^λ^**) < 1

or equivalently: r_w_∙x_1_ + λ_m_+ λ > 0 (b1)

From Equations (12) and (15) the values of (Zw_1_ = 0.31C) and (**b** = 0.538 – 0.179C) can be determined. We will define the exposure term for women (t_w_), at the exposure level (**x_1_**), to be the difference obtained by subtracting Equation (a1) from Equation (b1). Thus:

r_w_∙x_1_ – r_w_∙x_0_ = (r_w_)(x_1_ – x_0_) = t_w1_ > 0 (c1)

so that on a scale where (r_w_∙x_2_ – r_w_∙x_1_) is defined as equal to one unit (see Table 2), the point (x_0_) lies (t_w1_) units to the left of point (x_1_).

Similarly, from Equation (13), at an (x_1_) level of exposure yields:

[**a** – Zm_1_] / **a** = e^– (rm ∙ x1^ ^+^ **^λ^**^m)^ < 1

or equivalently: r_m_∙x_1_ + λ_m_ > 0 (d1)

From Equations (13) and (18), the values of (Zm_1_ = 0.085C) and (**a** = 0.1028 – 0.0496C) can be determined. Also, as was the case for Equation (c1), we will define the exposure term for men (t_m_), at the exposure level (**x_1_**), to be the difference obtained by subtracting a version of Equation (d1) evaluated at an (x_0_) exposure level from the same equation evaluated at the exposure level (**x_1_**). Note that, in this case, Equation (d1) evaluated at (**x_0_**) is (r_m_∙x_0_ + λ_m_ ≥ 0) and that (**x_0_**) need not be the threshold. Under these conditions:

r_m_∙x_1_ – r_m_∙x_0_ = (r_m_)(x_1_ – x_0_) = t_m1_ (e1)

Substituting the estimated values for **a**, **b**, Zw_1_, and Zw_2_ into the equations leading to (and including) Equation (22), using the derived upper bound (– 0.1) for Equation (22), and using the term (**λ**^true^) for the actual difference in threshold between men and women, this equation can be rewritten for the two different hazard-rates (r_w_ and r_m_) as:

r_w_∙x_1_ – r_m_∙x_1_ + λ^true^ = – ln{[**a/b**][(Zw_1_ – **b**) **/** (Zm_1_ – **a**)] } < – 0.1

or: (r_w_ – r_m_)x_1_ + λ^true^ = t_w1_ – t_m1_ < – 0.1 (f1)

We note that, because (x_1_ > 0), then (λ^true^ ≥ 0) if and only if [(r_w_ – r_m_) < –0.1]. Thus, men must have a lower threshold than women, a greater hazard-rate than women, or both. Both stated relationships cannot be false. Moreover, the term [(r_w_ – r_m_)x_1_] in Equation (f1) will be referred to as the “apparent” difference (λ^app^) in threshold levels between men and women at exposure level (**x_1_**) due to the difference in hazard-rate. Thus, the value of (**λ**) in Equation (b1) can be written as:

λ = λ_1_^app^ + λ^true^ < – 0.1

Note, from Equation (f1), that [λ^app^ = (r_w_ – r_m_)x_1_] and, thus, that (λ^app^) is proportional to the level of exposure (**x**).

In order to evaluate the potential magnitude of the difference in hazard-rate necessary for men to have the same (or a greater) threshold than women, we need to assess the apparent hazard for men at the exposure level (**x_1_**). Thus, Equation (d1) can be rewritten:

r_m_∙x_1_ + λ_m_ = r_w_∙x_1_ + (r_m_ – r_w_)x_1_ + λ_m_ = r_w_∙x_1_ – λ_1_^app^ + λ_m_ (g1)

Because the conditions of interest are (λ^true^ ≥ 0), then it follows from Equation (f1) that:

λ_1_^app^ < –0.1

For convenience, we will consider only the circumstance where (λ^true^ = 0) because the necessary conditions for (λ^true^ > 0) will only be more extreme. Also, we will use a translated coordinate system for the abscissa where (x' = x – x_0_), so that (x'_0_ = 0). Because, under the assumed conditions, men and women have the same “true” threshold (**x'_0_**), Equations (c1) and (e1) become:

r_w_∙x'_1_ – r_w_∙x'_0_ = (r_w_)(x'_1_) = t_w1_ (h1)

r_m_∙x'_1_ – r_m_∙x'_0_ = (r_m_)(x'_1_) = t_m1_ (i1)

dividing these two equations, and considering that (r_m_ > r_w_) from Equation (f1), this gives:

r_w_ / r_m_ = t_w1_ / t_m1_  < 1 (j1)

Because, by definition (Table 2), at the (**x_2_**) level of exposure, (r_w_∙x'_2_ = r_w_∙x'_1_ + 1) and from Equations (b1), (g1), (h1), and (i1), it follows that:

(r_w_)(x'_2_) = t_w1_ + 1 (k1)

(r_m_)(x'_2_) = (r_w_)(x'_2_) + (r_m_ – r_w_)x'_2_  = (t_w1_+1) – λ_2_^app^ (m1)

and dividing Equation (k1) by Equation (i1), yields:

(r_w_·x'_2_) / (r_m_·x'_1_) = (r_w_/r_m_)(x'_2_/x'_1_) = (t_w1_+1) / (t_m1_)

Therefore, by Equation (j1) this becomes:

(x'_2_/x'_1_) = [(t_w1_ + 1) / t_m1_] [t_m1_ / t_w1_] = (t_w1_+1)/ t_w1_ (n1)

Rewriting Equation (i1):

r_m_∙x'_1_ = t_w1_ + (r_m_ – r_w_)x'_1_ = t_w1_ – λ_1_^app^ (o1)

Therefore, from Equations (m1), (n1), and (o1):

(λ_2_^app^) / (λ_1_^app^) = [(r_w_ – r_m_)x'_2_] / [(r_w_ – r_m_)x'_1_] = (x'_2_/x'_1_) = (t_w1_+1)/ t_w1_ (p1)

And substituting Equation (o1) into Equation (p1) gives:

λ_2_^app^ = [(t_w1_ + 1) / t_w1_] [λ_1_^app^] (q1)

Therefore, as discussed in the Causal Model section above, because (0.25 < C < 0.76), then it must also be that (λ < – 0.1). In the present circumstance where (λ^true^ = 0), this means that (λ^app^ < – 0.1), which is the limit when (C = 0.25). Putting this into perspective, at (λ^app^ = – 0.1), then, at this boundary, (t_w1_ = 0.17), so that:

(λ_2_^app^)/(λ_1_^app^) = (x'_2_/x'_1_) = (t_w1_ + 1) / t_w1_) = 6.9

In the circumstance where (C = 0.76) and, thus, where (λ^app^ = – 4.6), then, at this boundary, (t_w1_ = 0.88), so that:

(λ_2_^app^)/(λ_1_^app^) = (x'_2_/x'_1_) = (t_w1_ + 1) / t_w1_) = 2.1

Consequently, between exposure-points (**x_1_**) and (**x_2_**), the parameter (λ^app^) should have changed by a factor of between 2.1 and 6.9 or, put another way, by a minimum of a factor of two over this interval. Importantly, therefore, the fact that it the estimate for (λ) doesn’t seem to change at all (Table 4) as one moves steadily (over time) from exposure-point (**x_1_**) to exposure-point (**x_2_**), supports the notion that the hazard-rate is approximately equal for the two genders. Consequently, even though a gender-specific difference in hazard-rate could explain some portion of the difference in threshold between men and women, it cannot explain it all (or even most of it). Thus, under any conceivable circumstances, men must have lower threshold of environmental exposure for getting MS compared to women.
